# Rupture post traumatique de la membrane de Bruch: à propos d'un cas

**DOI:** 10.11604/pamj.2015.21.314.7056

**Published:** 2015-08-28

**Authors:** Sanaa Ahbeddou, Jinane Ahmimeche, Nazih Tzili, Fadoua Alami, Ramzia Sebbah, Hamza Elorch, Amina Berraho

**Affiliations:** 1 Hôpital des Spécialités, Rabat, Maroc

**Keywords:** Traumatismes oculaires, rupture de la Bruch, néovaisseaux, eye injuries, Bruch rupture, neovascularization

## Abstract

Une contusion du globe peut se compliquer de rupture de la membrane de Bruch ou de la choroïde. Cette complication est observée dans 5 à 10% des cas avec une nette prédominance masculine. Nous rapportons l'observation clinique d'un patient de 26 ans, victime d'un traumatisme contusif sévère de l'œil gauche chez qui l'examen retrouve une rupture de la membrane de bruch au fond d'œil ; l'evolution spontanné a été marquée par une amélioration visuelle sans complications néovasculaires. Au cours des ruptures post traumatiques de la membrane de bruch le pronostic est essentiellement lié d'une part à sa localisation par rapport à la macula; et d'autre part à la survenue de complications néovasculaires (15 à 30 % des cas).

## Introduction

Les traumatismes oculaires contusifs sont un fréquent motif de consultation aux urgences ophtalmologiques ; toutes les structures oculaires peuvent être touchées. Nous rapportons l'observation clinique d'un jeune homme de 26 ans vu aux urgences ophtalmologiques du CHU Rabat, victime d'un traumatisme contusif sévère de l'œil gauche, par une barre en métal.

## Patient et observation

**Figure 1:**
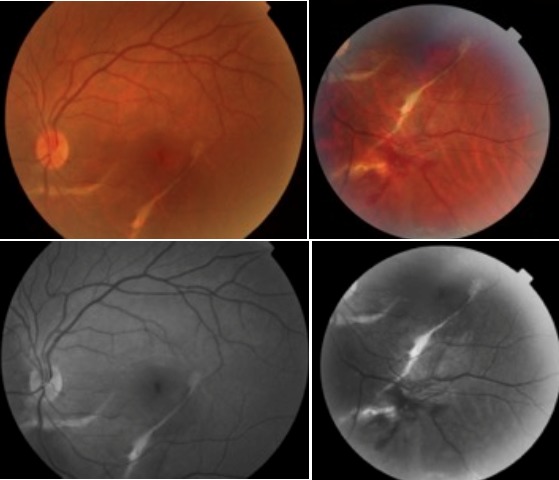
Photographie couleur et cliché anerythre montrant les lignes de rupture de la membrane de Bruch associée à des hémorragies prérétinienne et sous rétinienne inféro-maculaire et temporale inférieure

**Figure 2:**
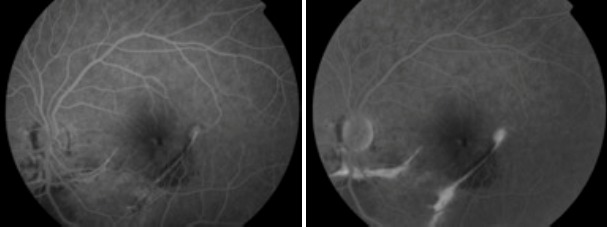
Hyperfluorescence linéaires spontanées sous papillaire et inféromaculaire, par effet fenetre sans diffusion au temps tardif fovéa épargnée à l'angiographie à la fuorésceine

**Figure 3:**
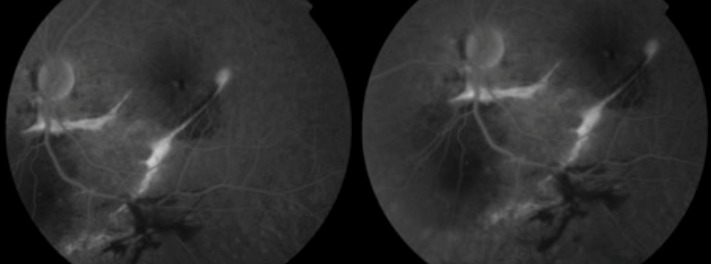
Hémorragies prérétinienne et sous rétinienne. Angiographie à la florésceine

**Figure 4:**
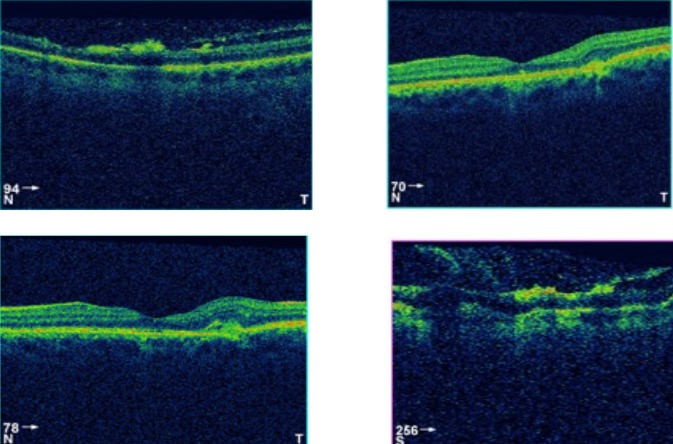
Coupe OCT passant par le centre de la zone de rupture avec conservation de la dépression fovéolaire

L'examen initiale trouve une acuité visuelle à 5/10 non améliorable (+0.25sph -0.25cyl 21°) P2 sans métamorphopsies. L'examen à la lampe à fente de l'œil gauche retrouve une semi mydriase réflexique. Le segment antérieur est normal. Le fond d'œil trouve de multiples lignes blanc-jaunâtres, au pôle postérieur et irradiant à partir de la papille; La papille et macula sans particularités (Figure 1). Le patient a bénéficié d'une angiographie rétinienne à la fluorescéine montrant un aspect d'hyperfluorescence linéaires spontanée sous papillaire et inféro- maculaire (Figure 2) et des hémorragies prérétiniennes et sous rétinienne inféro-maculaire et temporale inférieure (Figure 3). La tomographie en cohérence optique de l'œil gauche montre de multiples ruptures de la membrane de Bruch sans atteinte de la dépression fovéolaire ni aspect de néovaisseaux (Figure 4). L'examen de l'œil droit est normal. Lors des différents contrôles ophtalmologiques du suivi, l'évolution spontanée est marquée par la résorption progressive des hémorragies rétiniennes sans extension des lignes de rupture de la membrane de Bruch sur l'OCT,et sans complication néovasculaire, ainsi que la récupération d'une acuité visuelle à 8/10.

## Discussion

La rupture traumatique de l'épithélium pigmentaire de la rétine (EPR), de la membrane de Bruch, et de la choroïde survient dans 4 % à 10 % des contusions oculaires [1, 2]; avec une nette prédominance masculine sexe ratio 5/1 [3]. Le diagnostic est évoqué devant la nature du traumatisme (à globe fermé), l'aspect clinique: La rupture apparaît sous la forme d'un croissant jaunâtre, bien délimité, de disposition radiaire au nerf optique [1]; les données angiographique et OCT. L'angiographie au vert d'indocyanine trouve son intérêt dans les formes aiguës masquée par du sang ou en cas d'hémorragies ou la rupture parait sous forme d'une stries hypo fluorescente.

Le retentissement fonctionnel dépend du siège de la rupture; Près de la moitié des ruptures sont maculaires. Des ruptures multiples sont retrouvées dans 37 % des cas^1^, [4]. Il dépend également de la survenue d'une néovascularisation choroïdienne le plus souvent dans un délai de 1 à 37 mois [5] d'où l'intérêt d'une surveillance régulière du fond d'œil. Une néovascularisation doit être évoquée devant une BAV différé ou l'apparition de métamorphopsies. Le risque néovasculaire est plus élevé dans les six premiers mois nécessitant un recours au traitement par la photothérapie dynamique (PDT) ou l'injection d'anti-VEGF hors AMM selon les cas [6].

Dans notre cas, les lignes de rupture de la MB sont limitées au pôle postérieur avec respect de la dépression fovéolaire et sans complications néovasculaires sur 3 mois, ce qui explique la récupération spontanée d'une acuité visuelle correcte.

## Conclusion

La rupture de la membrane de Bruch est une complication fréquente des traumatismes oculaires post contusifs dont le risque est l'apparition des néovaisseaux choroïdiens. Une surveillance rigoureuse par angiographie à la fluorescéine est recommandée durant la première année.
